# Accouchement Force*Read at meeting of Austin District Medical Society, Austin, Texas, December, 1901.

**Published:** 1902-04

**Authors:** W. J. Matthews

**Affiliations:** Austin, Texas


For Texas Medical Journal.

### Accouchement Force.*

BY W. J. MATTHEWS, M. D., AUSTIN, TEXAS.

The subject I have chosen may not dazzle you as would the report of a daring and brilliant operation, or the report of some newly discovered chemical agent which promised to stay the onward destructive march of tuberculosis. But I take it that few of us will ever be called upon to perform great and serious operations, and perhaps it would be better for the glory of good surgery if fewer ordinary lights like ourselves attempted great operations. Again, during the last twenty years we have had so many cheering reports of remedies and methods which promised great things in the cure of pulmonary tuberculosis, but these remedies and methods have failed, and perhaps your hopes would be dissipated again by the report of another new remedy. So I bring before you today a common, useful, everyday subject with nothing visionary about it—a subject which deeply concerns us all every day of our lives. Per-

Read at meeting of Austin District Medical Society, Austin, Texas, December, 1901.

haps there is not before the writer today a single member of this society who has not stood by the bedside of a woman in the throes of labor in great anguish of mind asking himself earnestly what can I best do to terminate this long and agonizing labor with safety to mother and child? I trust we will all be helped to a solution of this question by a careful investigation of our subject, "Accouchement Force." By accouchement force are meant today three operations. First, complete instrumental or manual dilatation of the cervical canal, followed by second operation, either combined or direct version, or the application of the forceps, and, third, the immediate extraction of the child. The accouchement force of former days was often a dangerous and barbarous operation, followed by the most disastrous results. Without examining the condition of the cervix, the hand was forcibly thrust through an imperfectly dilated cervix and the fœtus immediately extracted through this imperfectly dilated canal. Is it any wonder, then, that rupture of the uterus was a common occurrence, followed by sad and fatal results? Even today in ordinary practice, safe and full dilatation of the cervix is not as often accomplished as it ought to be, as is proved by reports from our best maternity hospitals. In large cities emergency cases are often taken to these hospitals with the legs and body of the child born, and the head and arms still above the brim of the pelvis. You can imagine what force must be used to drag arms and head through such an imperfectly dilated os. What risks in such a case to the child's life and the mother's? Therefore, we would earnestly plead for complete dilatation of the cervix before extraction of the fœtus is attempted, and paralysis of the ring of the os also. You can tire out the uterine cervical muscle just as you can the rectal sphincter muscle, which, after full and prolonged dilatation, will remain dilated and paralyzed not only during an operation, but for days afterward. I shall only describe two methods of performing accouchement force. One of the methods, and a comparatively recent one, has been introduced by Dr. Philander A. Harris, of Patterson, N. J. The patient is thoroughly anesthetized. The index finger and thumb and the other fingers of the hand are successively inserted into the os, the thumb and fingers being spread apart as widely as possible. The thumb is kept extended whilst the other fingers are flexed. The drawing which I pass around will explain the method better than words can. This plan has been very successfully used by Dr. Harris and others. Another very excellent method is that recommended by Dr. Edgar, of New York. It is termed the bimanual method. It consists not only of complete dilatation with disappearance of the external cervical ring, but also in paralysis of the ring, so that the dangers of extraction, whether by forceps or version, may be reduced to the minimum for both mother and child. By this method you insert the index and middle finger of each hand into the cervix and forcible yet gradual and careful stretching is made until full dilatation with paralysis has been accomplished. The bimanual method is believed to be superior to other manual and instrumental methods for the following reasons: First, the membranes are preserved throughout the operation or until full dilatation is obtained. Second, there is no interference with the original presentation and position. Third, there is no constriction of the operator's hand. Fourth, the amount of force exerted upon the external ring can be better estimated, and hence there is less likelihood of lacerations occurring. Fifth, in placenta prævia there is less preliminary separation of the placenta by this method than by any other. Sixth, by no other method can not only complete dilatation, but also complete paralysis of the parturient os be so quickly and safely obtained. Before reporting my cases, I wish it distinctly understood that accouchement force as described in this paper is only intended for desperate emergency cases. That in the general run of obstetric practice it will seldom be required. Nevertheless, it is believed by the writer that there is a certain number of cases in which the lives of mother and child can be saved only by this method. In cases demanding accouchement force, we have to act and act quickly. The weapons of our warfare must always be on hand. Rubber dilaters might accomplish the work but rubber goods are perishable and may fail us just at a supreme critical moment. So in every way we prefer educated sensitive fingers to dilate the cervix to the most ingenious dilaters invented by human skill.

### REPORT OF CASES.

In March, 1895, I received a very urgent message to go five miles in the country to see a sick lady. After arriving at the place, I learned that the patient was within two weeks of her confinement. She had a high fever for twelve hours before my arrival. Her temperature at the time I examined her was 105° F. She was quite delirious. Movements of child had not been felt for two weeks. Careful auscultation failed to detect beat of fœtal. She had no chills and had felt fairly well up to the time she had fever. I considered my patient in a very grave condition and asked her husband to return to town and bring another physician with him. He quickly returned with my friend Dr. Bennett, who carefully examined patient and agreed with me that the child in utero was dead and that immediate delivery was in order. The cervix was not dilated and there was no effort at labor, but without any difficulty I could introduce my index finger into the cervix. I employed Dr. Harris's method in this case, which I knew best at this time. After thorough dilatation of cervix, I performed podalic version without any difficulty. The labor was begun and ended within one hour. The child was dead, confirming our diagnosis. The patient made an uneventful recovery.

Case 2.—This was a case of placenta praevia in which I helped one of our colored physicians. Just before I saw the case, the patient had a violent hemorrhage. Her pulse showed but too plainly the great loss of blood she had sustained. On examination I found that she had central implantation of the placenta. I followed the method recommended by Dr. Barnes. Introducing my hand into the vagina I separated the placenta all around the lower segment of the uterus as far as my index and middle fingers could reach. Very little bleeding followed this maneuvre. Then by the bimanual method I performed complete dilatation of the cervix. I then tried diligently to pass my fingers and hand by the border of the placenta, but it was adherent everywhere, so I was forced to pierce the placenta with my fingers in order to pass my hand into the uterus. As I was forcing my hand through the placenta the blood rushed furiously past my arm, but I quickly seized a foot turned and by traction on the foot brought the breech down to engage at the superior strait, causing strong pressure to be exerted on the placenta, thus rapidly arresting the much dreaded hemorrhage. In a short time the child was born. It was dead. I immediately delivered the placenta. The greatest trouble in the whole case was to arrest a threatening severe post-partem hemorrhage. The mother, after delivery, was very weak, exhausted and almost pulseless, but hypodermic injection of strychnia and the subcutaneous infusion of normal salt solution into the cellular tissue of subclavicular region, her heart's action quickly improved, and although she remained weak for sometime, she made a fairly rapid recovery. It is my firm belief that no other treatment would have saved this woman's life.

Case 3.—This case I attended recently. It was a primipara. It was also a normal head presentation. Slow labor pains continued for nearly forty-eight hours. By this time the woman was becoming rather exhausted and discouraged. Although her pains were quite severe for twenty-four hours, the os was at no time dilated more than the size of a half dollar. I gave as much chloral hydrate as I thought was safe. Gave chloroform occasionally, and used every effort to relax the cervix, but in vain. I told her husband that instrumental delivery was imperative, and that I wanted professional assistance. He sent for Dr. Goodall Wooten. Dr. Wooten, after examining the case, agreed with me that instrumental delivery was the proper procedure, but advised rupturing the membranes and waiting for four hours, hoping that the pains would be augmented and possibly natural delivery effected. After the lapse of four hours if the pains were not stronger and better dilatation of cervix was not obtained, Dr. Wooten was to be recalled, and immediate delivery accomplished by whatever method should be deemed best. At the end of four hours the pains were not improved, and the os was positively less dilated than at last examination, and the temperature was now 101° F. Dr. Wooten was recalled, and we were perfectly agreed that forcible dilatation of the cervix and a high forceps operation were our only alternatives. Dr. Wooten put the patient completely under chloroform. By the bimanual method I thoroughly dilated and paralyzed the cervix. I then applied the forceps at the brim. It was a difficult forceps operation, as the head of the child had not engaged at the brim, but my able assistant so steadied the uterus by well directed manual pressure that I got a firm hold of the head of the fœtus with the forceps. After one hour's long, hard work the child was delivered alive, and, I am happy to state, without any laceration of the woman's perineum. The child's head was severely bruised, but today it shows no evidence of the pressure of the forceps. The patient recovered without a rise of temperature, and with very little traumatic pain afterwards. In all these cases it is almost unnecessary to state that the most rigid antiseptic precautions were used. The external genitals were thoroughly scrubbed with green soap and hot water. The vagina was irrigated with a creolene solution of proper strength. I scrubbed my hands for five minutes with green soap and boiled water, then cleansed them with boiled water. Then I immersed them in a strong solution of permanganate of potash, and then in a strong solution of oxalic acid, and finally immersed them in a 1 to 1000 of the bichloride of mercury solution before introducing my hand into vagina or uterus. This treatment of the hands takes time, but it is time well spent, and is always a life-saving process.

